# Understanding the impact of haemodialysis on UK National Health Service patients’ well‐being: A qualitative investigation

**DOI:** 10.1111/jocn.13871

**Published:** 2017-07-03

**Authors:** Daniel JW Jones, Kate Harvey, John P Harris, Laurie T Butler, Emma C Vaux

**Affiliations:** ^1^ School of Psychology University of Reading Malaysia Nusajaya Johor Malaysia; ^2^ School of Psychology and Clinical Language Sciences University of Reading Reading Berkshire UK; ^3^ Department of Renal Medicine Royal Berkshire NHS Foundation Trust Reading UK

**Keywords:** chronic kidney disease, haemodialysis, psychological well‐being, psychology, qualitative study

## Abstract

**Aims and objectives:**

While haemodialysis is an effective treatment for end‐stage renal disease, the requirements and restrictions it imposes on patients can be onerous. The aim of this study was to obtain UK National Health Service patients’ perspectives on the challenges arising from haemodialysis with the intention of identifying potential improvements.

**Background:**

Depression rates are particularly high in those with end‐stage renal disease; however, there is limited insight into the range of stressors associated with haemodialysis treatment within the National Health Service contributing to such high rates, particularly those of a cognitive or psychological nature.

**Design:**

A qualitative approach was used to obtain rich, patient‐focused data; one‐to‐one semi‐structured interviews were conducted with twenty end‐stage renal disease at a UK National Health Service centre.

**Methods:**

Patients were interviewed during a typical haemodialysis session. Thematic analysis was used to systematically interpret the data. Codes were created in an inductive and cyclical process using a constant comparative approach.

**Results:**

Three themes emerged from the data: (i) fluctuations in cognitive/physical well‐being across the haemodialysis cycle, (ii) restrictions arising from the haemodialysis treatment schedule, (iii) emotional impact of haemodialysis on the self and others. The findings are limited to predominantly white, older patients (median = 74 years) within a National Health Service setting.

**Conclusions:**

Several of the experiences reported by patients as challenging and distressing have so far been overlooked in the literature. A holistic‐based approach to treatment, acknowledging all aspects of a patient's well‐being, is essential if optimal quality of life is to be achieved by healthcare providers.

**Relevance to clinical practice:**

The findings can be used to inform future interventions and guidelines aimed at improving patients’ treatment adherence and outcomes, for example, improved reliable access to mental health specialists.

**What does this study contribute to the wider global clinical community?**

- Provides a comprehensive understanding of the physiological, emotional, social and psychological challenges faced by haemodialysis patients in the UK National Health Service (NHS).
- Highlights areas of patient care in which future interventions could be targeted to improve treatment adherence and patient outcome.

## 1 INTRODUCTION

Haemodialysis (HD) is a crude instrument. While it can prolong life, it is unable to replicate the complexities of the human renal system. Its requirements in terms of time and proximity to a specific treatment centre begin immediately and continue without respite. Patients endure physical distresses associated with HD, such as fatigue (Liu, 2006), pain (Verhallen, Kooistra, & van Jaarsveld, 2007), restrictions to food and fluid intake (Baraz, Parvardeh, Mohammadi, & Broumand, 2010) and reduced physical activity (Johansen et al., 2000). Its constraints impinge on holidays, social activities, employment and socio‐economic status (Chilcot, Wellstead, Da Silva‐Gane, & Farrington, 2008; Norris & Agodoa, 2005; Rodriguez et al., 2007). Evidence also indicates a detrimental impact on marital and family relationships (Soskolne & De‐Nour, 1987). Depression rates are high among end‐stage renal disease (ESRD) patients (Kalender, Ozdemir, Dervisoglu, & Ozdemir, 2007; Martin, Tweed, & Metcalfe, 2004) but studies demonstrating rates increase with duration of HD suggest that poorer psychological well‐being may be better explained by the challenges arising from the HD treatment regime than the actual disease (Kimmel, Thamer, Richard, & Ray, 1998; Lopes et al., 2002). In laboratory studies of cognition, ESRD has been associated with multiple impairments (Hart & Kreutzer, 1988), for example, of visual attention (Etgen et al., 2009), executive functioning (Jassal, Devins, Chan, Bozanovic, & Rourke, 2006) and psychomotor speed (Griva et al., 2003). In addition, various aspects of memory have been shown to be impaired: verbal and visual memory (Elias et al., 2009; Kurella, Chertow, Luan, & Yaffe, 2004), working memory (Buchman et al., 2009), episodic memory (Thornton, Shapiro, Deria, Gelb, & Hill, 2007) and conceptual memory (Jones et al., 2015b). However, it is uncertain whether patients are aware of these impairments, and to what extent they impact on everyday life. There are also suggestions that cognitive performance fluctuates around the HD schedule, but the evidence for this is mixed. Thus in the study of Griva et al. (2003), patients were tested once immediately preceding and once 24 hr after dialysis. The authors reported significant improvements in attention, concentration, verbal and visual memory and psychomotor speed, postdialysis. In contrast, Murray et al. (2007) examined patients global cognitive functioning at four different times around a dialysis session: 1 hr before, 1 hr into a session, 1 hr after and the following day. The authors found that cognitive performance was worst during dialysis, with performance improving 1 hr after dialysis. Patients performed at their best 1 hr before and the following day after dialysis, with no significant difference between the scores at these times, unlike in the study of Griva et al. 2003. It is not clear at present why this difference between studies arose.

Several studies have used qualitative methods to explore aspects of the impact of ESRD from the patient's perspective. Examining factors underlying the patient's choice of treatment, Morton, Devitt, Howard, Andersson, Snelling, & Cass, (2010a) and Morton, Tong, Howard, Snelling, & Webster, (2010b) find a strong focus on autonomy with overtreatment as opposed to effectiveness of treatment or life expectancy being key. Ashby et al. (2005) describe the factors which influence some patients to stop treatment altogether; these include impaired quality of life and the desire not to burden others. In a Swedish study, the main areas of reported suffering came from the loss of freedom involved in HD and the dependence on caregivers (Hagren, Pettersen, Severinsson, Luetzen, & Clyne, 2001). Patients felt that their lives were restricted by the need for HD and that it consumed much of their time. They also felt that there was an emotional distance between themselves and the doctors and nurse caring for them and experienced a feeling of vulnerability (Hagren, Pettersen, Severinsson, Luetzen, & Clyne, 2005). The theme of inability to communicate effectively with staff in renal units, both before dialysis started (and so its burdens came as a shock) and later in treatment when patients’ health may have deteriorated, emerged in a UK study (Bristowe et al., 2015). In a Greek study (Theofilou, Synodinou, & Panagiotaki, 2013), data from semi‐structured interviews indicate that HD is associated with unemployment, functional disturbance, nonadherence to medication and diet, social isolation, fatigue, psychological distress and sexual dysfunction. Moreover, they reveal implications that may not be considered by clinicians, for example, the social isolation experienced as a result of HD scheduling constraints. Some of the findings may be specific to the Greek healthcare system, which has been adversely affected by recent austerity measures (Karamanoli, 2011), and to cross‐cultural differences in other studies. Nevertheless, loss of freedom, sometimes poor communication between professionals and patients, and (potentially serious [Denhaerynck et al., 2007]) nonadherence to treatment are also found among National Health Service (NHS) patients (Karamanidou, Weinman, & Horne, 2014). Spiers and Smith (2016) also highlight the accompanying stressors associated with waiting for a kidney transplant (a position that many HD patients are in). The main stressors identified include the uncertainty of waiting for a kidney and receiving a transplant from a living donor; patients reported that these lead to an additional sense of confusion and worry alongside the HD treatment process itself. It is noteworthy that a recent review of studies of quality of life in ESRD (Joshi, 2014) concluded that many relevant domains were currently omitted, including thinking, learning, memory concentration, patients’ feelings about their health, dependence on treatments and the financial burden of treatments. A related point was made by Morris, Biggerstaff, and Lycett (2016), who urged that listening to patients’ voices is important in plans for improving renal health care.

The aim of this study was to better understand some of the challenges faced by ESRD patients undergoing thrice‐weekly HD in a hospital‐based renal unit, especially those noted by Joshi to be omitted from most studies of ESRD. A qualitative approach was selected because it provides rich, meaningful, patient‐focused data. It enables problematic and/or distressing aspects of HD, perhaps overlooked by clinicians, to be identified and delineated. It has been used effectively for this purpose with other chronic illness populations, namely cancer (Ashing‐Giwa et al., 2004), diabetes (Rubin & Peyrot, 1999), stroke (Lynch et al., 2008) and dementia (Steeman, Casterlé, Dierckx, Godderis, & Grypdonck, 2006). Qualitative studies investigating the impact of ESRD have provided substantial, useful data; however, because one of the aims of qualitative research was to provide understanding of the meanings and interpretations of behaviour within the specific sample under study, it is of value to conduct further qualitative research with additional samples in order to establish the extent to which similar themes emerge and new themes are revealed. In this study, we were particularly interested in the fluctuations in cognitive abilities caused by HD, reported to us by patients in quantitative studies of cognition (Jones, Butler, Harris, & Vaux, 2015a; Jones, et al., 2015b). In addition, this study aims to provide an understanding of the patient experience in the UK NHS. Being the largest and oldest publicly funded healthcare system in the world, the NHS is a unique facility and, although many experiences are likely to be shared across other healthcare settings, due to the scale and composition of the organisation, there may be some issues/concerns that are specific to the NHS.

## 2 METHODS

The study was approved by an NRES Committee and by a University Research Ethics Committee and was conducted by the principal investigator (PI) who designed the study, conducted the interviews and coded the data. The study was designed and conducted in line with Guba and Lincoln's criteria for rigour in qualitative research (Denzin & Lincoln, 1994) and reported using the COREQ checklist (Tong, Sainsbury, & Craig, 2007).

### 2.1 Design

For this study, one‐to‐one semi‐structured interviews were conducted with ESRD patients, and the data were analysed using content analyses. ESRD patients were recruited from two renal NHS units located in the UK. One unit is an in‐hospital dialysis centre located in a large general hospital. The other unit is a dialysis‐only unit located at a single site independent from any hospital. All patients were receiving thrice‐weekly HD for 3–5 hr per treatment and had been receiving HD for a minimum of 90 days prior to testing (adequate dialysis at Kt/v > 1·4). All patients meeting these criteria (*n* = 60) were sent an invitation letter from their consultant nephrologist outlining that the purpose of the interview was to gain an insight into how dialysis patients manage their treatment and the effects it has on daily activities. The PI met patients once they had expressed an interest in the study. At this meeting, PI described that the research was part of a doctorate thesis examining the impact of renal disease on cognition and quality of life, and explained the study in greater detail. PI interviewed all those who agreed to participate, providing a consecutive sample. As well as being practical, this approach maximised the diversity of the sample by enabling any patient meeting the criteria to participate. Interviews were conducted with 20 patients: eight male and 12 female whose ages ranged from 55–88 years with a median of 74. One of the 20 interviews was terminated prematurely due to the interviewee feeling unwell. No one approached declined to participate. Towards the end of interviewing, the researcher noted that little new data were appearing and concepts were well developed (theoretical saturation). Several additional interviews were conducted to confirm this impression. Given that consecutive sampling had maximised participant diversity, and that the data obtained had been systematically compared within and between participants, the researchers judged that after the twentieth interview, sufficient data had been collected to address the research question.

### 2.2 Data collection

Individual semi‐structured interviews were conducted by the PI following training from an experienced qualitative researcher. Written informed consent was obtained prior to interview. Interviews were conducted at the bedside during a typical HD session. The location of interviews is important in qualitative research as no setting is truly neutral and all settings shape the data. The bedside was selected because it was convenient for both researcher and patient, and because it avoided attrition. Its disadvantages were that patients may have felt inhibited by the presence of other patients and healthcare practitioners, or perceived the research to be connected to treatment. The researcher was careful to ensure patients consented to being interviewed at the bedside and that they could not be overheard. The researcher also explained that the research was not connected to treatment. However, it is acknowledged that patients’ accounts were influenced by the setting. A topic guide (see Appendix A) comprising twelve open‐ended questions was used; it was developed from a review of ESRD literature and from informal conversations with patients and nursing staff on an NHS dialysis ward. Patients were informed that the researcher was not part of the clinical team and was interested in understanding the impact of HD on all aspects of the patient's life, for example: “Do you find there are tasks, which are quite familiar to you, that you find more difficult now than before you were ill?” and “How would you rate your overall memory?” The topic guide was used flexibly, and the interviewer used prompts and probes to encourage patients to expand and elaborate on topics, and to introduce any additional concerns or issues they considered relevant. Each interview lasted between 13–42 min (median length: 23 min) and was stopped when patients felt they had nothing further to add. Interviews were audio‐recorded and transcribed verbatim. Detailed field notes were kept.

### 2.3 Data analysis

Transcripts were analysed using the Atlas.ti v.7 software package (Atlas.ti Scientific Software Development GmbH, Berlin, Germany). Thematic analysis was used to group common ideas across transcripts allowing generation of higher and lower codes (labels representing an idea or theme). A systemised approach to coding was used (Fereday & Muir‐Cochrane, 2006), and the principals of the constant comparison Method in which data are coded and re‐coded iteratively and inductively were employed (Strauss & Corbin, 1994). Coding began by noting and grouping common patterns, themes or metaphors that encapsulated general ideas. Next, initial codes were revised and regrouped to create more definitive groups, which primarily involved making further connections between different categories or topics. Finally, common categories were finalised and unified around a central theme. Quotes were then identified and linked to the appropriate unifications to form clusters of quotes to represent codes. The quotes presented in results section were drawn from these clusters and selected because they best represented a code or illustrated different aspects of a code. Codes were then combined to generate families of co‐occurring ideas or similar overarching themes. For example, the codes “predialysis problems,” “postdialysis problems” and “problems during dialysis” were united to form the family called “fluctuations in cognitive and physical well‐being across the dialysis cycle.” Coding was undertaken by the PI and discussed with several other researchers so that internal thinking processes were made explicit, ideas clarified and new insights obtained.

## 3 RESULTS

Three distinct themes emerged from the data:

1. Fluctuations in cognitive and physical well‐being across the HD cycle.
2. Restrictions arising from the HD treatment schedule.
3. Emotional impact of HD on the self and others.

### 3.1 Fluctuations in cognitive and physical well‐being over the dialysis cycle

Cognitive fluctuations were classified as problems with mental processes such as fluidity of thought, concentration and memory, whereas physical fluctuations were classified as somatogenic symptoms such as pain or fatigue. Multiple patients expressed concerns about their memory; many described poor short‐term memory, specifically remembering to carry out day‐to‐day tasks. A commonly described occurrence was the inability to accurately remember names of people from the past and new people they had interacted with on the ward (e.g., nurses):

P4, male, 56 years: *“…I think my memory is not as good as it was three plus years ago; generally it's not quite as good.” and “…it has been a concern a bit that, you know, some things I think, “Oh I should have remembered that, such and such” and you don't, some people's names.”*

One patient explicitly associated their memory loss with their HD treatment but the majority attributed it to old age or medication. Few believed their memory problems fluctuated with the dialysis schedule, describing general, rather than time specific, memory deficiency:

P4, male, 56 years: *“I suppose regularly after dialysis not being with it as much, I suppose my memory is almost, it becomes lazy. Err, yeah, it's probably a bit worse, it's almost certainly worse after dialysis.”*

P8, male, 84 years: *“My memory has, normally due to my age I think my memory has lost a bit, lost a bit of its sharpness. I don't remember things very well, and err, but the dialysis hasn't changed that.”*

However, several described an impact on their physical and cognitive health. This appeared to be irrespective of age. Three distinct groups emerged:

- Patients who were physically and cognitively fatigued by HD.
- Patients who were physically but not cognitively fatigued by HD.
- Patients who were unaffected by HD.

For patients in Group A, there was a notable difference in well‐being between dialysis and nondialysis days. These patients reported mental fatigue, drowsiness, tiredness, light headedness and a lack of concentration and motivation after a typical dialysis session, which resulted in them being unable to undertake simple day‐to‐day activities and had an impact on employment. P1 described the effect as that of “…a fly being swatted…you feel dopey” (P1, female, 73 years). Patients in this group quite often reported wanting to sleep after dialysis rather than engage in anything more cognitively demanding such as reading or completing a crossword. Two patients reported a disruption to their fluidity of thought, impairing conversations with nurses and transport staff. The cognitive and physical impact of HD was especially problematic for this group of patients if they were dialysed in the morning, several of whom described a feeling of “wasted days”:

P1, female, 73 years: “*Well you're a bit dopey if you know what I mean. I come off far dopier than I went on, which to a certain extent annoys you, because I come here to have this treatment but I feel worse when I have it.”* and *“…I don't want to concentrate, I don't want to be bothered, you don't want to be bothered, I just want it shut off, I just sit in my chair and go to sleep.”*

P6, female, 82 years: *“…go down and the bus drivers start chatting, “Oh, you alright?”, and I can't think of an answer, because the brain doesn't work, it just sort of, everything has gone from you. You're just a person.”*

Patients in Group A typically reported their cognitive functioning was restored to normal, and their physical tiredness greatly reduced, by the day after HD. Indeed, nondialysis days were generally regarded as better for their cognitive and physical well‐being, and an opportunity to carry out activities or socialise. However, this type of reaction was not unique to Group A patients but was also observed in Group B patients:

P11, male, 77 years: *“Oh the best time is the day after dialysis when you've got a free day, or the weekend…”*

The distinctive feature of Group B patients was that the physical fatigue experienced following HD was not accompanied by an impact on cognitive functioning. Like those in Group A, when patients in Group B returned home following treatment most wanted to sleep and found activities such as watching television or reading resulted in falling asleep. They were also unable to undertake activities or socialise postdialysis. Unlike Group A patients, they perceived this as a physical, not cognitive, response to HD:

P8, male, 84 years: *“No, I feel generally physically tired, that's all. But mentally I am alright”*

P5, female, 66 years: *INT: “You feel mentally fatigued as well?”, P: “No, not really, just overall tiredness. By about 8 o'clock tonight I'll be back to sleep, that's my day gone really.”*

As with Group A, by the day following HD, Group B patients were more alert and could undertake normal daily activities. In contrast to Group A and Group B patients, Group C patients reported no effect on their cognitive or physical functioning following HD. Consequently, their accounts do not feature the perception of HD as being restrictive or time‐consuming, as commonly described by Group A and Group B patients:

P18, male, 75 years: “*I don't notice any difference from when I come on dialysis to when I go off dialysis.”*

P3, female, 59 years: *“And normally when I get home I can do just about anything, you know, and if I start to feel a little bit funny I'll drink something and just replenish the fluid a little bit.”*

The emergence of three distinct groups demonstrates the heterogeneity of HD patients postdialysis and provides a better sense of the variation between individuals within a single treatment system. Of note is the different post‐treatment capacity that patients report with some able to engage with a healthcare professional after HD, whereas for others this is too challenging.

The general consensus of the sample was that the time period immediately preceding dialysis is one of optimal well‐being—it is the treatment procedure itself that causes a decrease in well‐being. Patients typically stated that they felt no different in advance of dialysis, for example: “No, no. It's just like a normal day, the only difference is I come for dialysis instead of go out shopping.” (P15, male, 70 years). An alternative perspective was provided by a handful of patients who described feeling adverse effects of a long period without dialysis. While they were unable to articulate the feeling precisely, they described feeling below par. However, there was no discernible pattern to this response; patients reporting these predialysis experiences did not correspond to any specific postdialysis group (A, B or C). This suggests that there may be greater heterogeneity pre‐HD compared to the post‐HD experience:

P4, male, 56 years: *“Sometimes on a Monday, following a weekend without the dialysis, I feel almost as if I'm needing the dialysis. I don't know if it's psychological or physiological, or a bit of both, but I feel it's time; I need to have it done.”* and *“it starts on the Sunday really, um, the day before I have dialysis, at about mid‐afternoonish I start to feel yes it would be a good idea to have dialysis…”*

P4, male, 56 years: *“On a score out of ten, I would say 8 out of 10 now [on dialysis], compared with, as I said about the Sunday, starting to feel as though you need dialysing, that's probably about 2 to 3 out of 10.”*

### 3.2 Restrictions arising from the HD treatment schedule

A second theme that emerged from interviews was the practical limitations arising from HD. Many patients commented on the stress caused by the various requirements of HD, the time‐consuming nature of dialysis (upwards of 14 hr per week) and the permanency of the treatment process without a definitive endpoint. The combination of these factors is unique to patients receiving HD.

The most common restriction patients reported related to holidays and going on vacation. At one stage or another, all were prevented from travelling or going on holiday (either abroad or in the UK) by their treatment regime, and almost all commented that this had an impact on their psychological well‐being, causing frustration and in some cases despondency:

P6, female, 82 years: *“Oh yeah, and I think that's why I am probably depressed because I'm not getting a holiday. I haven't had a holiday for three years.”*

P5, female, 66 years *“Well that is frustrating, cos we used to go away, at least once a year abroad, and course can't do that now, so I can't get no sunshine.”*

Some patients expressed disappointment about the impact of HD on their retirement plans (the majority of patients in the sample were approaching retirement age or already retired). They described anticipating their retirement as a time to undertake longed‐for activities, but finding the restrictions associated with HD made those aspirations unattainable:

P4, male, 56 years: *“Oh and before I had this condition I thought that when I retired I would do a lot of travelling and all that sort of thing, um, that's out of the picture, that's not going to happen.”*

A specific restriction, mentioned by the majority of participants, related to diet. If patients’ salt, glucose, alcohol, fluid intake and exercise are not controlled, “fluid overload” can ensue, resulting in breathlessness, swelling and hypertension, potentially leading to cardiovascular problems and possible death (Franz, Pohanka, Tribl, Woloszczuk, & Hörl, 1997). Between dialysis sessions, patients are given tailored programmes specifying fluid, food and sodium intake. Their weight is monitored before and after dialysis sessions, and they are required to monitor their dietary intake constantly and without respite. They are unable to indulge even occasionally and are advised not to drink alcohol. The majority of patients described eventually acclimating to these restrictions, although most found doing so difficult:

P11, male, 77 years: INT: *“Your diet is quite restricted?”* P11: *“Yeah that was initially a problem but I seem to have got the mental adjustment to it now, like not to drink too much…”*

However, a minority responded differently to the dietary restrictions recommended. These patients confessed to ignoring the guidance of their treating dieticians and following their own diet.

P2, female, 69 years: *“Supposed to be yeah, but I'm afraid most of the time I ignore it. Most of the foods they recommend in here I don't like. So I don't stick to my diet very well, which the doctor doesn't like.”*

While the long‐term impact of this decision is probably detrimental to physical health outcomes (Morduchowicz et al., 1993), exercising this freedom of choice appeared to have some benefits for patients in terms of reduced stress:

P18, male, 75 years: *“Well they do tell you in here because there's a dietician, don't have this and don't have that. No, I have a normal diet to be honest and it works.”*

A major concern spontaneously voiced by many participants was the restrictions arising from NHS provided transport to and from their dialysis sessions, which was described as frequently and sometimes substantially delayed. For patients, this meant already long treatment sessions were further extended. For example, *P14* reported that on one occasion, following dialysis lasting several hours, they had to wait a further 2 hr for his transport home, culminating in a treatment session lasting 8 hr. Participants reported anger and frustration at about the time‐consuming transport delays, particularly as they further curtailed already restricted days, increasing stress:

P13, female, 80 years: *“And sometimes you have to wait quite a while, it's the waiting that's the trouble, you know, you feel tired and just want to go home and you have to wait around for somebody who's not finished.”*

P15, male, 88 years: *“Sometimes we have to wait a hell of a time for it. One classic night there was three of us, live near each other, we were all picked up together, we had to wait until 7 o'clock, and er, not happy about that. It lengthens your day and you're fed up and you think “Why are we waiting here?” and there's no real answer.”*

P16, female, 63 years: *“Sometimes I finish at half 4, then I have to wait until quarter to 6. Again I'm a very impatient person, by then I am always angry.”*

Sacrificing employment or job opportunities because of HD was common among patients, whether it was retiring, working part‐time or limiting working hours to fit in with the dialysis schedule. For those patients still employed, this had a financial impact, but importantly was also described as a challenge to their work ethic:

P4, male, 56 years: *“So, yeah, the spin‐off will affect my income, which in a way I feel a bit grieved about, I don't think it should, but it's going to. But I can't, I don't think I can do too much about it. But I'm talking with them at the moment, so we'll see how it goes.”*

Retirees expressed frustration at not having the freedom to complete chores and undertake daily activities such as housework and gardening. They frequently commented that HD restricted the amount of time they have to undertake such jobs; the more severely affected patients had only four available days per week; partly due to HD, but also because of the adverse effects of treatment. In addition to restricting the time available for daily chores, patients also described HD as restricting their time available for socialising. Consequently, patients considered their usable time had become more valuable and sacred:

P4, male, 56 years: *“It is wasting my life, every Monday, Wednesday, [and Friday] I certainly wouldn't go to evening classes, definitely wouldn't, wouldn't touch it with a barge pole. I used to occasionally go to evening classes over my lifetime, but not after dialysis, there'd be no point, I get absolutely no enjoyment, I'd hate being there, and I probably wouldn't remember a lot. There'd be absolutely no point, be a waste of money.”*

P1, female, 73 years: *“Cos you do, well you got no life, you might think it's coming in having it, but it's not you haven't got no life. You have, 3 days a week, then doctors’ appointments, one Dr, this Dr, that Dr, you're literally being pulled apart if you know what I mean.”*

Once again, these examples demonstrate the variability across HD patients, in that stressors are very much dependent upon the lifestyle expectations of individual patients. However, what is apparent across the group is the consistent reports of frustrations and unhappiness at the overall impact that treatment has upon their quality of life.

### 3.3 Emotional impact of HD on the self and others

A third theme that emerged from the interviews was the impact of HD on patients’ emotional health and the impact this had on relationships. Patients described being unable to spend as much time with family members and friends as they wished, in addition to the strain on relationships arising from the restrictions of the treatment schedule:

P4, male, 56 years: *“My daughter lives over in South Wales. You know, I'm a bit wary; I wouldn't go and spend a few days there because I've got dialysis here. I know you can arrange things, but I don't really want to be going through that palaver in doing that, so perhaps, I don't know, I see her less because of this, but if I do take this slightly early retirement the opportunity to see her more won't necessarily go up as more as if I hadn't been on dialysis, so that's another issue.”*

P11, male, 77 years: *“…cos I've got a lot of friends scattered around the place and I don't get to see them.”*

Patients also reported concern that their HD was a constant worry and stress for their family members. Typically, clinicians and support groups focus on the impact of HD on patients, but this study highlights the considerable impact the disease, and treatment, has on their family, in particular the practical and emotional strain placed on them and the impact on their psychological well‐being:

P17, female, 70 years: *“Well my husband sorts out all my medication, drives him mad it does because I've got so much different stuff that I take, and er, he does that night and day. He looks after me in the house as well…”*

P12, female, 79 years: *“My husband couldn't take it in, I don't think he can now; he goes to pieces if anything unforeseen happens to me.”*

P7, female, 81 years: *“I feel a bit sorry for my husband because, you know, he probably would have to like to go to Greece or something, we've been there a few times.”*

Throughout the interviews, patients frequently described an emotional response to specific aspects of HD, for example, the depressing effect of restrictions to travel, but beyond these, there was a more overarching emotional response. Numerous patients discussed feelings of helplessness, describing themselves as victims of the treatment, rather than the treatment benefiting them. Many patients described themselves as being in a fragile emotional state due to the physical and emotional demands of treatment, and several reported depression and resentment. Patients reported wanting, on occasion, to abandon treatment because it was so challenging to persevere with it week after week:

P2, female, 69 years: *“…actually 2 weeks ago I was on the verge of giving it up, I didn't want to do dialysis anymore. My family nagged me and now I am back on dialysis. I resent the fact that…before I came on dialysis they said: “Once you're on dialysis you will feel fine, you won't know you've got kidney problems, you'll feel fine. They lied to me, they don't know because they are not on dialysis. It does affect you, it affects your life. You get very depressed.”*

P1, female, 73 years: *“They said well, they more or less said to me every year about you're going to die, but I have told them I don't want to do this for the rest of my life, I know it might be only 7–10 years at the most, I just don't want to do it. I don't want to do this for the rest of my life. I just, I just can't explain it, it's just not nice, you feel like your body is being invaded, all the time.”*

P2, female, 69 years: *“I just resent being on dialysis, I just don't want to be here. If someone said I could go now I would go. I suppose because it's changed my life I resent it. The fact that I'm on dialysis 3 days a week four hours a day.”*

The longevity of HD treatment was exposed as a major factor in patients’ emotional state. Patients reported that one of the hardest parts of the treatment process is the multiple unknowns: they do not know how long the treatment will last, whether they will ever be rid of HD and whether they will ever get a transplant. Consequently, they viewed HD as a never‐ending process from which they could not escape, which lead to feelings of frustration, resentment and depression:

P6, female, 82 years: *“But I just like my freedom, and it just ties you down completely. And I wouldn't mind if there was some end, say in three years’ time or so you'll be free, but it's just the rest of your life.”*

P2, female, 69 years: *“Well yeah, I got a good lecture off my family and Jean told me off. Couldn't see…I just thought why am I still coming here, I'm not going to be better in 6 weeks’ time or 6 months’ time, I'll still have it. I will probably be worse than what I am now; can't see an end to it.”*

While many patients were negative towards HD several tried to focus on its positive aspects and, by acknowledging the alternative to HD was death, these patients appeared able to achieve a degree of acceptance. For some patients, age contributed to their more positive outlook: these patients described feeling gratitude for the life they had had, and viewed HD as enabling them to further extend it:

P9, female, 87 years: *“For me, I'm 87 this year, so there is no transplant or anything. So that is true, so you must help yourself by listening to what you are told and learning about yourself as much as you can, but you've got to understand your body and understand your illness, and to listen to what you are told and do your very best.”*

P7, female, 81 years: *“…what you have got to think of is this is keeping me alive; I'd be dead, I mean years ago a lot of people died if you had kidney failure. So umm, I'm very pleased in that respect.”*

P8, male, 84 years: *“In the beginning yes until I got used to it, I am quite used to it now, I am happy to come. I come here three times a week and it consumes, the day's absolutely lost. I start at 12.30 and finish at 5.30–6.00, so the day is gone.”*

Having considered themes individually and in‐depth, we then considered the relationships between them. The three themes described the effect of HD on distinct and specific aspects of patients’ lives, but together, they also reflect the cumulative nature of these stressors. Although stressors are personal, based partly on age and lifestyle, their collective impact was shared among all patients. In particular, patients described how the impact of HD on one aspect of life encroached on other aspects of life, for example, one patient reflected how the inability to be spontaneous affected their emotional health:

P6, female, 82 years: *“Umm, also you find it difficult when you'd love to say “Oh yes I'll go off to get the bus to Newbury”. But you can't do that I'm at RBH tomorrow for dialysis. And that sort of gets depressing, makes you depressed.”*

The cumulative and encroaching impact of HD on one's well‐being was summed up by P4:

P4, male, 56 years: *“It affects spouse, one's spouse. It affects so many different things, and those all have a depressing effect.”*

### 3.4 Summary of key findings

1. Fluctuations in cognitive and physical well‐being across the HD cycle: In terms of the cognitive and physical effects of the treatment, patients have a heterogeneous experience of HD. This investigation highlights the variation in patient experience; healthcare staff are unable to provide support with a one‐size‐fits‐all approach.
2. Restrictions arising from the HD treatment schedule: Irrespective of age and lifestyle, restrictions, particularly relating to vacations, work and transport, have a considerable negative impact on patient quality of life.
3. Emotional impact of HD on the self and others: An often overlooked issue, HD puts a considerable strain on personal relationships, and consequently impairing patient quality of life. Patients described feelings of helplessness, viewing HD as detrimental to their overall well‐being, with several patients stating episodes of depression and resentment towards the treatment.

## 4 DISCUSSION

Semi‐structured interviews were conducted with twenty patients with ESRD receiving thrice‐weekly HD at NHS centres. Three core themes emerged which described (i) the impact of HD on patients’ cognitive and physical well‐being, (ii) the restrictions arising from the treatment schedule and (iii) the emotional impact of HD on the self and others.

Fluctuations in physical and mental well‐being coinciding with the schedule of dialysis treatment were reported by many patients. Although fluctuations in physical comfort have been associated with fluid levels over the course of the dialysis cycle (Jaeger & Mehta, 1999), the implications and exact manifestations of this discomfort have yet to be fully explored. Although others have noted that some patients experience unpleasant symptoms at the end of a dialysis session (Karamanidou et al., 2014), this study is the first to identify categories of response to HD; three distinct groups, consistent in their differences over time, emerged from the data (physically and mentally affected, only physically affected, unaffected), demonstrating the range of patient demands, within one treatment setting, that renal healthcare professionals need to be aware of. Further research is required to determine whether these categories are generalisable to the broader population of patients. If they are, clinicians may be able to use them to predict which patients are more susceptible to adverse physical and mental effects and so target additional support, perhaps by tailoring patients’ HD schedules and avoiding early HD sessions for patients adversely affected. The existence of subgroups of patients whose performance may be affected differently by dialysis suggests one possible explanation for the inconsistency in the studies of the effects of HD on cognition. Clearly, if the majority of patients in a study are those who have reported no effects or only physical effects of dialysis, then the chances of finding an effect on cognition are reduced. The reasons why patients are differentially affected by HD are not clear. Biochemical changes have been suggested as one explanation for such differences; a study by Griva, Thompson, Jayasena, Davenport, Harrison, & Newman, (2006) found weak correlations between neuropsychological performance and biochemical measures. Madan, Kalra, Agarwal, and Tandon (2007) also found a positive correlation between serum creatinine, blood urea and uric acid with P3 latency (an early measure of processing speed and thus cognitive impairment). Findings of this relationship are somewhat limited though, and thus inconclusive. What is clear, however, is that the reliable adverse effects of HD on the cognitive state of some patients need to be taken into account when choosing when to give important information about treatment.

Several aspects of our data echo the findings of other studies. The most common concern among this group of patients with ESRD related to the restrictions associated with HD. In many interviews, patients described a typical dialysis week as arduous and resulting in a loss of independence with the impact extending to partners and family. Strict scheduling of HD prevents patients from having a regular job, impacting upon income and consequently the ability to support a family, as also found by Hagren et al. (2005). As in our study, these authors also noted that the amount of time spent on dialysis and adverse effects of treatment restrict the time available for daily activities. Although small in isolation, the accumulation of such restrictions has a detrimental effect on overall well‐being and patient's quality of life. This study raises the awareness to clinicians and healthcare managers that the restrictions patients face, while seemingly minor, are accumulative. Demonstrating a greater understanding and willingness to find creative and bespoke solutions, where possible, is likely to have a positive impact. Patients need to be acknowledged as experts in their illness and treatment. They should be supported to identify effective ways in which they might cope with the impacts of HD specific to them. Time needs to be taken to understand the specific challenges HD raises for individual patients so that care can be tailored to better address their needs. Petrie and Weinman (2012) advocate the idea of supporting patients to fully comprehend their illness, and their perceptions towards it, leading to better outcomes. The upshot for patients with ESRD would be better treatment adherence, happier patients and carers who can continue to lend support.

Dietary restrictions appeared to be particularly problematic, such that some patients rejected clinicians’ advice, risking the significant health implications of poor ESRD management (Kalantar‐Zadeh & Kopple, 2001; Locatelli et al., 2002). Others have noted that this is especially problematic in the case of fluid intake when patients become thirsty (Hagren et al., 2005). However, while patients often confess to eating forbidden foods, skipping medication appears to be less deliberate (Karamanidou et al., 2014), more likely a consequence of the cognitive deficits associated with ESRD (Jones et al., 2015a). It is important for clinicians to be aware that when patients perceive the restrictions on diet and fluid intake to be unacceptably onerous, they abandon them. There is a need for strategies to be developed that seek to improve patients’ perceived control and ability to implement advice; once again, interventions such as those advocated by Petrie and Weinman (2012), at changing perceptions, through guidance, to fully understand their illness.

A key issue that emerged from the data related to the NHS transport system. A patient may take advantage of the free transport provided by the NHS, but ever‐increasing demands on the system may mean a wait for transport to arrive. For patients who already feel that their independence has been curtailed, the addition of an avoidable (at least perceived) extended period in hospital (sometimes for periods of 2 hr or more) as a consequence of delays amplified frustration and anxiety. An Australian‐based study (Morton et al., 2010a, 2010b) identified one of the negative perceptions of hospital‐based dialysis was the need to travel. A multinational study, including the UK, conducted by Moist et al. (2008) suggested that a longer one‐way travel time to the dialysis centre contributed to lower quality of life and a significantly greater risk of mortality in patients. Given the impact of transport problems on NHS HD patients, continued attempts to improve the service would likely have a substantial positive impact on patients.

Personal relationships were noted as a significant factor influencing patient well‐being. The reliance, worry and stress put on a family carer can manifest as anger and frustration, significantly impacting on patients. The extra burden of care on family members has been seen by patients as a negative aspect of home‐based dialysis (Morton et al., 2010b). Evidence suggests that the stresses of caring for a relative can have a negative impact on the immune system, making the carer more vulnerable to infectious diseases (Vedhara et al., 1999). Alvarez‐Ude, Valdes, Estébanez, and Rebollo (2003) found that caregivers of patients on dialysis experienced a higher burden, had a worse score on a quality of life measure (HRQOL) and had a higher risk of clinical depression than the normal population of a similar age. Auer (2002) suggests it is imperative to address the carer's needs, as well as the patient's, as much of a patient's time is spent away from the hospital. If we are to consider patient welfare holistically, with the end goal being optimum quality of life and prolonged life, then carer welfare is equally important in this equation. Current estimations suggest that nine of 10 carers of HD patients are close relatives and that the presence of a family carer improves patients’ treatment adherence and compliance to dietary restrictions (Cicolini, Palma, Simonetta, & Di Nicola, 2012). The cancer literature highlights the importance of the practical, emotional and psychological support that caregivers provide for patients (Thomas, Morris, & Harman, 2002) while the stroke literature suggests that family caregivers often feel isolated and that their own needs are neglected, leading to strains on the relationship (McKevitt, Redfern, Mold, & Wolfe, 2004). It is important for clinicians and healthcare managers in the renal setting to recognise the vital care relatives provide so that they can be supported to continue in their caregiving role.

A dichotomy arose between patients that had a generally negative outlook and those who had a positive one; a possible explanation being a patient's subjective locus of control. Christensen, Turner, Smith, Holman, and Gregory (1991) investigated the relationship between locus of control and depression in patients with ESRD. The extent to which patients believed that their health was controllable was directly linked to whether they had recently received a failed transplant or not, with those that had experiencing greater rates of depression than those who had not. The authors suggest that perception of control contributes significantly to patient outcome when dealing with patients with chronic illness. In this study, those patients who viewed HD as a choice in order to prolong life, and something that is under their control, generally tended to have a more positive outlook. If control and autonomy, with relation to the disease or treatment, can be addressed, then the benefit to patients’ emotional well‐being may be considerable. Introducing brief in‐hospital interventions that increase patients’ autonomy is likely to improve the holistic experience of the treatment process, facilitating better patient outcome. However, further investigations are required to establish a clear association between locus of control and patient outlook in the HD population.

The experiences described here are those of patients receiving HD treatment within an NHS hospital setting, and therefore, some of the concerns and issues raised may not be relevant for patients receiving peritoneal dialysis or HD treatment in other institutions. Furthermore, although a diverse sample of HD patients was sought, the sample was predominantly white and over 70 years of age. Although reflecting the typical patient receiving HD in an NHS setting, the concerns of other groups may not have been captured. For example, children receiving HD may have a different experience of treatment compared to an older adult population. Further studies using a similar methodology with other groups should be conducted so that their experiences can be better understood.

## 5 CONCLUSION

The use of semi‐structured interviews enabled the discussion of a broad range of issues directly relevant to HD patients. Some of these issues, such as transport concerns and fluctuations in cognition, have so far been overlooked in the ESRD literature. By documenting patients’ experiences in a systematic fashion, we can begin to highlight areas that, if addressed, have the potential of a substantial impact on patient welfare. However, as noted from this investigation, this requires a clear, holistic approach, with a joint effort from clinicians, care providers and healthcare managers. In addition, investigations such as these can be used to educate new patients who are naïve to the demands of HD treatment. It is clear that there are patients in the NHS who, prior to treatment, were not made aware of the internal and external stressors that accompany HD treatment (P2, female, 69: “They lied to me, they don't know because they are not on dialysis. It does affect you, it affects your life. You get very depressed”). Curtin and Mapes (2001) highlighted education and self‐management of treatment as essential tools to improving overall patient well‐being.

## 6 RELEVANCE TO CLINICAL PRACTICE

The issues highlighted in this study are relevant to all those involved in the treatment of ESRD in an NHS setting, including the HD patients themselves. Through semi‐structured interviews, patients have provided a rich account of the practical and emotional challenges that accompany HD. The data suggest that a holistic approach to treatment and care is required. Potential areas for improvement have been identified, for example, the introduction of additional support networks, including the introduction of easy access to an established psychology and/or social work service for all NHS patients. These findings can be used to inform such future interventions and guidelines, to establish a framework with the aim to improve patients’ holistic experience of HD, adherence to treatment and medication, and overall outcome. Given the complexity of patients’ experiences, and the heterogeneity of patient demands, further studies trialling the feasibility and acceptability of such interventions are required. One area of investigation may be to look at the role of perceived control on medication and dietary adherence. Although the data were gathered within an NHS setting, there is no obvious rationale for why many of the suggestions for improvements in treatment could not be applied equally to other healthcare systems.

## CONTRIBUTIONS

Study design: DJ, JH, LB, EV data collection and analysis: DJ; and manuscript preparation: DJ, KH, JH, LB, EV.

## References

[jocn13871-bib-0001] Alvarez‐Ude, F. , Valdes, C. , Estébanez, C. , & Rebollo, P. (2003). Health‐related quality of life of family caregivers of dialysis patients. Journal of Nephrology, 17(6), 841–850. 15593060

[jocn13871-bib-0002] Ashby, M. , Op't Hoog, C. , Kelleher, A. , Kerr, P. G. , Brooks, D. , Nicholls, K. , & Forrest, M. (2005). Renal dialysis abatement: Lessons from a social study. Palliative Medicine, 19, 389–396. 10.1191/0269216305pm1043oa 16111062

[jocn13871-bib-0003] Ashing‐Giwa, K. T. , Padilla, G. , Tejero, J. , Kraemer, J. , Wright, K. , Coscarelli, A. , … Hills, D. (2004). Understanding the breast cancer experience of women: A qualitative study of African American, Asian American, Latina and Caucasian cancer survivors. Psycho‐Oncology, 13(6), 408–428. 10.1002/pon.750 15188447 PMC1618782

[jocn13871-bib-0004] Auer, J. (2002). Dialysis—a family matter. A personal tribute to the relatives of kidney patients. EDTNA/ERCA Journal, 28(3), 141–144. 10.1111/j.1755-6686.2002.tb00229.x 12371739

[jocn13871-bib-0005] Baraz, S. , Parvardeh, S. , Mohammadi, E. , & Broumand, B. (2010). Dietary and fluid compliance: An educational intervention for patients having haemodialysis. Journal of Advanced Nursing, 66(1), 60–68. 10.1111/j.1365-2648.2009.05142.x 20423436

[jocn13871-bib-0006] Bristowe, K. , Horsley, H. L. , Shepherd, K. , Brown, H. , Carey, I. , Matthews, B. , … Murtagh, F. E. (2015). Thinking ahead‐the need for early Advance Care Planning for people on haemodialysis: A qualitative interview study. Palliative Medicine, 29, 443–450. 25527527 10.1177/0269216314560209 PMC4404401

[jocn13871-bib-0007] Buchman, A. S. , Tanne, D. , Boyle, P. A. , Shah, R. C. , Leurgans, S. E. , & Bennett, D. A. (2009). Kidney function is associated with the rate of cognitive decline in the elderly. Neurology, 73, 920–927. 10.1212/WNL.0b013e3181b72629 19657107 PMC2754333

[jocn13871-bib-0008] Chilcot, J. , Wellstead, D. , Da Silva‐Gane, M. , & Farrington, K. (2008). Depression on dialysis. Nephron Clinical Practice, 108, 256–264. 10.1159/000124749 18401193

[jocn13871-bib-0009] Christensen, A. J. , Turner, C. W. , Smith, T. W. , Holman, J. M. , & Gregory, M. C. (1991). Health locus of control and depression in end‐stage renal disease. Journal of Consulting and Clinical Psychology, 59(3), 419 10.1037//0022-006X.59.3.419 2071727

[jocn13871-bib-0010] Cicolini, G. , Palma, E. , Simonetta, C. , & Di Nicola, M. (2012). Influence of family carers on haemodialyzed patients’ adherence to dietary and fluid restrictions: An observational study. Journal of Advanced Nursing, 68(11), 2410–2417. 10.1111/j.1365-2648.2011.05935.x 22360845

[jocn13871-bib-0011] Curtin, R. B. , & Mapes, D. L. (2001). Health care management strategies of long‐term dialysis survivors. Nephrology Nursing Journal: Journal of the American Nephrology Nurses’ Association, 28(4), 385. 12143460

[jocn13871-bib-0100] Denhaerynck, K. , Manhaeve, D. , Dobbels, F. , Garzoni, D. , Nolte, C. , & De Geest, S. (2007). Prevalence and consequences of nonadherence to hemodialysis regimens. American Journal of Critical Care 16, 222–235. 17460313

[jocn13871-bib-0012] Denzin, N. K. , & Lincoln, Y. S. (1994). Handbook of qualitative research. Thousand Oaks, CA: Sage Publications, Inc.

[jocn13871-bib-0013] Elias, M. F. , Elias, P. K. , Seliger, S. L. , Narsipur, S. S. , Dore, G. A. , & Robbins, M. A. (2009). Chronic kidney disease, creatinine and cognitive functioning. Nephrology Dialysis Transplantation, 24, 2446–2452. 10.1093/ndt/gfp107 PMC2727297 19297357

[jocn13871-bib-0014] Etgen, T. , Sander, D. , Chonchol, M. , Briesenick, C. , Poppert, H. , Forstl, H. , & Bickel, H. (2009). Chronic kidney disease is associated with incident cognitive impairment in the elderly: The INVADE study. Nephrology Dialysis Transplantation, 24, 3144–3150. 10.1093/ndt/gfp230 19461010

[jocn13871-bib-0015] Fereday, J. , & Muir‐Cochrane, E. (2006). Demonstrating rigor using thematic analysis: A hybrid approach of inductive and deductive coding and theme development. International Journal of Qualitative methods, 5(1), 80–92. 10.1177/160940690600500107

[jocn13871-bib-0016] Franz, M. , Pohanka, E. , Tribl, B. , Woloszczuk, W. , & Hörl, W. H. (1997). Living on chronic hemodialysis between dryness and fluid overload. Kidney International Supplement, 59, S39–S42. 9185103

[jocn13871-bib-0017] Griva, K. , Newman, S. P. , Harrison, M. J. , Hankins, M. , Davenport, A. , Hansraj, S. , & Thompson, D. (2003). Acute neuropsychological changes in hemodialysis and peritoneal dialysis patients. Health Psychology, 22, 570–578. 10.1037/0278-6133.22.6.570 14640853

[jocn13871-bib-0018] Griva, K. , Thompson, D. , Jayasena, D. , Davenport, A. , Harrison, M. , & Newman, S. P. (2006). Cognitive functioning pre‐to post‐kidney transplantation—a prospective study. Nephrology Dialysis Transplantation, 21(11), 3275–3282. 10.1093/ndt/gfl385 16861731

[jocn13871-bib-0019] Hagren, B. , Pettersen, I.‐M. , Severinsson, E. , Luetzen, K. , & Clyne, N. (2001). The haemodialysis machine as a lifeline: Experiences of suffering from end‐stage renal disease. Journal of Advanced Nursing, 34(2), 196–202. 10.1046/j.1365-2648.2001.01745.x 11430281

[jocn13871-bib-0020] Hagren, B. , Pettersen, I.‐M. , Severinsson, E. , Luetzen, K. , & Clyne, N. (2005). Maintenance haemodialysis: Patients’ experiences of their life situation. Journal of Clinical Nursing, 14, 294–300. 10.1111/j.1365-2702.2004.01036.x 15707439

[jocn13871-bib-0021] Hart, R. P. , & Kreutzer, J. S. (1988). Renal system In Tarter R. E., Van Thiel D. H., & Edwards K. L. (Eds.), Medical neuropsychology: The impact of disease on behaviour (pp. 99–120). New York, NY: Plenum Press.

[jocn13871-bib-0022] Jaeger, J. Q. , & Mehta, R. L. (1999). Assessment of dry weight in hemodialysis: An overview. Journal of the American Society of Nephrology, 10(2), 392–403. 10215341 10.1681/ASN.V102392

[jocn13871-bib-0023] Jassal, S. V. , Devins, G. M. , Chan, C. T. , Bozanovic, R. , & Rourke, S. (2006). Improvements in cognition in patients converting from thrice weekly hemodialysis to nocturnal hemodialysis: A longitudinal pilot study. Kidney International, 70, 956–962. 10.1038/sj.ki.5001691 16837916

[jocn13871-bib-0024] Johansen, K. L. , Chertow, G. M. , Ng, A. V. , Mulligan, K. , Carey, S. , Schoenfeld, P. Y. , & Kent‐Braun, J. A. (2000). Physical activity levels in patients on hemodialysis and healthy sedentary controls. Kidney International, 57(6), 2564–2570. 10.1046/j.1523-1755.2000.00116.x 10844626

[jocn13871-bib-0025] Jones, D. J. W. , Butler, L. T. , Harris, J. P. , & Vaux, E. C. (2015a). Latent learning in end stage renal disease (ESRD). Physiology and Behaviour, 142, 42–47. 10.1016/j.physbeh.2015.01.033 25637651

[jocn13871-bib-0026] Jones, D. J. W. , Harris, J. P. , Vaux, E. , Hadid, R. , Kean, R. , & Butler, L. T. (2015b). The nature of impairments of memory in patients with end‐stage renal disease (ESRD). Physiology & Behaviour, 147, 324–333. 10.1016/j.physbeh.2015.05.008 25980628

[jocn13871-bib-0027] Joshi, V. D. (2014). Quality of life in end stage renal disease. World Journal of Nephrology, 3(4), 308–316. 25374827 10.5527/wjn.v3.i4.308 PMC4220366

[jocn13871-bib-0028] Kalantar‐Zadeh, K. , & Kopple, J. D. (2001). Relative contributions of nutrition and inflammation to clinical outcome in dialysis patients. American Journal of Kidney Diseases, 38(6), 1343–1350. 10.1053/ajkd.2001.29250 11728973

[jocn13871-bib-0029] Kalender, B. , Ozdemir, A. C. , Dervisoglu, E. , & Ozdemir, O. (2007). Quality of life in chronic kidney disease: Effects of treatment modality, depression, malnutrition and inflammation. International Journal of Clinical Practice, 61, 569–576. 10.1111/j.1742-1241.2006.01251.x 17263698

[jocn13871-bib-0030] Karamanidou, C. , Weinman, J. , & Horne, R. (2014). A qualitative study of treatment burden among haemodialysis recipients. Journal of Health Psychology, 19(4), 556–569. 10.1177/1359105313475898 23471760

[jocn13871-bib-0031] Karamanoli, E. (2011). Debt crisis strains Greece's ailing health system. The Lancet, 378(9788), 303–304. 10.1016/S0140-6736(11)61152-5 21789785

[jocn13871-bib-0032] Kimmel, P. L. , Thamer, M. , Richard, C. M. , & Ray, N. F. (1998). Psychiatric illness in patients with end‐stage renal disease. The American Journal of Medicine, 105(3), 214–221. 10.1016/S0002-9343(98)00245-9 9753024

[jocn13871-bib-0033] Kurella, M. , Chertow, G. M. , Luan, J. , & Yaffe, K. (2004). Cognitive impairment in chronic kidney disease. American Geriatrics Society, 52, 1863–1869. 10.1111/j.1532-5415.2004.52508.x 15507063

[jocn13871-bib-0034] Liu, H. E. (2006). Fatigue and associated factors in hemodialysis patients in Taiwan. Research in Nursing & Health, 29(1), 40–50. 10.1002/nur.20109 16404733

[jocn13871-bib-0035] Locatelli, F. , Fouque, D. , Heimburger, O. , Drüeke, T. B. , Cannata‐Andía, J. B. , Hörl, W. H. , & Ritz, E. (2002). Nutritional status in dialysis patients: A European consensus. Nephrology Dialysis Transplantation, 17(4), 563–572. 10.1093/ndt/17.4.563 11917047

[jocn13871-bib-0036] Lopes, A. A. , Bragg, J. , Young, E. , Goodkin, D. , Mapes, D. , Combe, C. , … Port, F. K. (2002). Dialysis Outcomes and Practice Patterns Study (DOPPS): Depression as a predictor of mortality and hospitalization among hemodialysis patients in the United States and Europe. Kidney International, 62(1), 199–207. 10.1046/j.1523-1755.2002.00411.x 12081579

[jocn13871-bib-0037] Lynch, E. B. , Butt, Z. , Heinemann, A. , Victorson, D. , Nowinski, C. J. , Perez, L. , & Cella, D. (2008). A qualitative study of quality of life after stroke: The importance of social relationships. Journal of Rehabilitation Medicine, 40(7), 518–523. 10.2340/16501977-0203 18758667 PMC3869390

[jocn13871-bib-0038] Madan, P. , Kalra, O. P. , Agarwal, S. , & Tandon, O. P. (2007). Cognitive impairment in chronic kidney disease. Nephrology Dialysis Transplantation, 22(2), 440–444. 10.1093/ndt/gfl572 17023495

[jocn13871-bib-0039] Martin, C. R. , Tweed, A. E. , & Metcalfe, M. S. (2004). A psychometric evaluation of the Hospital Anxiety and Depression Scale in patients diagnosed with end‐stage renal disease. British Journal of Clinical Psychology, 43, 51–64. 10.1348/014466504772812968 15005906

[jocn13871-bib-0040] McKevitt, C. , Redfern, J. , Mold, F. , & Wolfe, C. (2004). Qualitative studies of stroke a systematic review. Stroke, 35(6), 1499–1505. 10.1161/01.STR.0000127532.64840.36 15105517

[jocn13871-bib-0041] Moist, L. M. , Bragg‐Gresham, J. L. , Pisoni, R. L. , Saran, R. , Akiba, T. , Jacobson, S. H. , … Port, F. K. (2008). Travel time to dialysis as a predictor of health‐related quality of life, adherence, and mortality: The Dialysis Outcomes and Practice Patterns Study (DOPPS). American Journal of Kidney Diseases, 51(4), 641–650. 10.1053/j.ajkd.2007.12.021 18371540

[jocn13871-bib-0042] Morduchowicz, G. , Sulkes, J. , Aizic, S. , Gabbay, U. , Winkler, J. , & Boner, G. (1993). Compliance in hemodialysis patients: A multivariate regression analysis. Nephron, 64(3), 365–368. 10.1159/000187355 8341381

[jocn13871-bib-0043] Morris, A. , Biggerstaff, D. , & Lycett, D. (2016). Capturing whole patient care. Journal of Renal Care, 42(2), 71–72. 10.1111/jorc.12158 27145903

[jocn13871-bib-0044] Morton, R. L. , Devitt, J. , Howard, K. , Andersson, K. , Snelling, P. , & Cass, A. (2010a). Patient views about treatment of Stage 5 CKD: A qualitative analysis of semistructured interviews. American Journal of Kidney Disease, 55(3), 431–440. 10.1053/j.ajkd.2009.11.011 20116914

[jocn13871-bib-0045] Morton, R. L. , Tong, A. , Howard, K. , Snelling, P. , & Webster, A. C. (2010b). The views of patients and carers in treatment decision making for chronic kidney disease: Systematic review and thematic synthesis of qualitative studies. British Medical Journal, 340, 10.1136/bmj.c112 PMC2808468 20085970

[jocn13871-bib-0046] Murray, A. M. , Pederson, S. L. , Tupper, D. E. , Hochhalter, A. K. , Miller, W. A. , Qi Li, M. S. , … Foley, R. N. (2007). Acute variation in cognitive function in hemodialysis patients: A cohort study with repeated measures. American Journal of Kidney Diseases, 50(2), 270–278. 10.1053/j.ajkd.2007.05.010 17660028

[jocn13871-bib-0047] Norris, K. C. , & Agodoa, L. Y. (2005). Unravelling the racial disparities associated with kidney disease. Kidney International, 68(3), 914–924. 10.1111/j.1523-1755.2005.00485.x 16105022

[jocn13871-bib-0048] Petrie, K. J. , & Weinman, J. (2012). Patients’ perceptions of their illness: The dynamo of volition in health care. Current Directions in Psychological Science, 21, 60–65. 10.1177/0963721411429456

[jocn13871-bib-0049] Rodriguez, R. A. , Sen, S. , Mehta, K. , Moody‐Ayers, S. , Bacchetti, P. , & O'Hare, A. M. (2007). Geography matters: Relationships among urban residential segregation, dialysis facilities, and patient outcomes. Annals of Internal Medicine, 146(7), 493–501. 10.7326/0003-4819-146-7-200704030-00005 17404351

[jocn13871-bib-0050] Rubin, R. R. , & Peyrot, M. (1999). Quality of life and diabetes. Diabetes/Metabolism Research and Reviews, 15(3), 205–218. 10.1002/(SICI)1520-7560(199905/06)15:3<c205:AID-DMRR29>e3.0.CO;2-O 10441043

[jocn13871-bib-0051] Soskolne, V. , & De‐Nour, A. K. (1987). Psychosocial adjustment of home hemodialysis, continuous ambulatory peritoneal dialysis and hospital dialysis patients and their spouses. Nephron, 47(4), 266–273. 10.1159/000184522 3696328

[jocn13871-bib-0052] Spiers, J. , & Smith, J. A. (2016). Waiting for a kidney from a deceased donor: an interpretative phenomenological analysis. Psychology, Health & Medicine, 21, 836–844. 10.1080/13548506.2015.1112415 26584590

[jocn13871-bib-0053] Steeman, E. , Casterlé, D. , Dierckx, B. , Godderis, J. , & Grypdonck, M. (2006). Living with early‐stage dementia: A review of qualitative studies. Journal of Advanced Nursing, 54(6), 722–738. 10.1111/j.1365-2648.2006.03874.x 16796664

[jocn13871-bib-0054] Strauss, A. , & Corbin, J. (1994). Grounded theory methodology. Handbook of Qualitative Research, 17, 273–285.

[jocn13871-bib-0055] Theofilou, P. , Synodinou, C. , & Panagiotaki, H. (2013). Undergoing haemodialysis: A qualitative study to investigate the lived experiences of patients. Europe's Journal of Psychology, 9(1), 19–32.

[jocn13871-bib-0056] Thomas, C. , Morris, S. M. , & Harman, J. C. (2002). Companions through cancer: The care given by informal carers in cancer contexts. Social Science & Medicine, 54(4), 529–544. 10.1016/S0277-9536(01)00048-X 11848273

[jocn13871-bib-0057] Thornton, W. L. , Shapiro, R. J. , Deria, S. , Gelb, S. , & Hill, A. (2007). Differential impact of age on verbal memory and executive functioning in chronic kidney disease. Journal of International Neuropsychological Society, 13, 344–353. 10.1017/S1355617707070361 17286891

[jocn13871-bib-0058] Tong, A. , Sainsbury, P. , & Craig, J. (2007). Consolidated criteria for reporting qualitative research (COREQ): A 32‐item checklist for interviews and focus groups. International journal for quality in health care, 19(6), 349–357. 17872937 10.1093/intqhc/mzm042

[jocn13871-bib-0059] Vedhara, K. , Cox, N. K. , Wilcock, G. K. , Perks, P. , Hunt, M. , Anderson, S. , … Shanks, N. M. (1999). Chronic stress in elderly carers of dementia patients and antibody response to influenza vaccination. The Lancet, 353(9153), 627–631. 10.1016/s0140-6736(98)06098-x 10030328

[jocn13871-bib-0060] Verhallen, A. M. , Kooistra, M. P. , & van Jaarsveld, B. C. (2007). Cannulating in haemodialysis: Rope‐ladder or buttonhole technique? Nephrology Dialysis Transplantation, 22(9), 2601–2604. 10.1093/ndt/gfm043 17557776

