# Supplementary material for: Understanding the impact of haemodialysis on UK National Health Service patients’ well‐being: A qualitative investigation
Source: J Clin Nurs. 2017 Jul 3;27(1-2):193–204. doi: 10.1111/jocn.13871 (PMC6853155; doi:10.1111/jocn.13871)
Supplement: Supplementary file 1 [file JOCN-27-193-s001.docx]

## Appendix A – Semi-structured interview topic guide

Structured Interview

**Introduction (5 minutes)**

*Introduce yourself, the project and research theme. Explain the facilitator’s role and the objective of the research. Explain what use will be made of audio recordings. Explain that the participant is free to express their opinion, that their opinion matters, that there are no right or wrong answers and that this should be enjoyable.*

**Blurb:**

Thank you very much for taking part in this interview. The overall objective of this discussion is to learn more about how dialysis affects individuals’ day-to-day lives. Taking part in this interview allows us to understand more about what it is like to live with dialysis and the scale of the effect it has on your life. Don’t worry about speaking openly as there are no right or wrong answers to the questions I’ll be asking you. The session will be recorded, but all the data from the session will be kept confidential and the identity of individuals will not be revealed to anyone other than the administrators of the focus group.

**Questions**

1. Do you find there are tasks, which are quite familiar to you, that you find more difficult now than before you were ill?

- *Chores*
- *Telephone use*
- *Cooking etc.*

1. Over the course of your weekly dialysis cycle do you notice any fluctuations in your ability to carry out tasks? Differences between pre and post dialysis?

- *When?*
- *Due to tiredness or inability to concentrate etc.*
- *Reading a book (pre or post)?*

1. (If they do drive) Do you find that driving has become more challenging since you became ill?

(If they don’t drive) Has travelling independently become more challenging since you were ill?

- *Navigation*
- *Concentration*
- *Attention*

1. Do you still go out as much now as before you were ill? Still keep in touch with old friends?

- *Tolerance*
- *Patience dealing with others (younger children etc.).*

1. How would you rate your ability to keep track of day-to-day things, for example, bills, medication?

- *Remembering birthdays*
- *Time management*
- *Hospital appointments*

1. Since you became ill do you find that you now become more easily tired?

- *Sleep more*
- *Can no longer walk long distances*

1. Has your mood changed at all since you became ill? Do you suffer from mood swings?

- *First diagnosed, how did you feel?*
- *Offered counselling?*
- *Personality changes*

1. Are there any aspects of shopping that you have difficulty with since you became ill?

- *Shopping lists*
- *Payment process*

1. Do you still enjoy talking to others? Do you ever forget names/the names of objects?
2. How would you rate your overall memory?

- *Short-term*
- *Long- term*
- *Ever get confused / anxious?*

1. Since you became ill, do you feel that you have to rely on someone else more so now, than before?

- *Carer? / Partner? / Friends?*
- *Pressures on you and your partner’s relationship?*

1. Are there any other difficulties you may have that you would be willing to discuss?

**Feedback and close**

*Summarise main points of discussion and ask for their feedback.* The project, of which the interviews form an important part, will enable better understanding of how dialysis affects individuals’ day-to-day lives. Thank you for attending the interview and the contribution you have made. If any respondents require further information about the nature of the research being undertaken or require any clarification on how these discussions will be used please contact: xxxxxxxxxxxxx
